# Adipose tissue metabolic changes in chronic kidney disease

**DOI:** 10.1097/IN9.0000000000000023

**Published:** 2023-04-27

**Authors:** Eurico Serrano, Prashamsa Shenoy, Maria Paula Martinez Cantarin

**Affiliations:** 1 Division of Nephrology, Department of Medicine, Sidney Kimmel Medical College at Thomas Jefferson University, Philadelphia, PA, USA

**Keywords:** chronic kidney disease, adipose tissue inflammation, adipokines, chronic inflammation, protein energy wasting, insulin resistance, adiponectin, leptin

## Abstract

Adipose tissue is a complex organ whose functions go beyond being an energy reservoir to sustain proper body energy homeostasis. Functioning as an endocrine organ, the adipose tissue has an active role in the body’s metabolic balance regulation through several secreted factors generally termed as adipokines. Thus, adipose tissue dysregulation in chronic kidney disease (CKD) can have a deep impact in the pathophysiology of diseases associated with metabolic dysregulation including metabolic syndrome, insulin resistance (IR), atherosclerosis, and even cachexia. CKD is a progressive disorder linked to increased morbidity and mortality. Despite being characterized by renal function loss, CKD is accompanied by metabolic disturbances such as dyslipidemia, protein energy wasting, chronic low-grade inflammation, IR, and lipid redistribution. Thus far, the mechanisms by which these changes occur and the role of adipose tissue in CKD development and progression are unclear. Further understanding of how these factors develop could have implications for the management of CKD by helping identify pharmacological targets to improve CKD outcomes.

## 1. Introduction

Chronic kidney disease (CKD) is one of the most common organ dysfunctions with a rising prevalence in the last decade ^[1,2]^. CKD patients suffer from increased atherosclerosis, chronic inflammation, malnutrition, insulin resistance (IR), and other metabolic derangements that contribute to significant morbidity and mortality ^[3–7]^.

Renal function impairment leads to the systemic accumulation of multiple metabolic waste compounds, commonly designated as uremic toxins, which have adverse effects on body function. These toxins include degradation products of protein metabolism (asymmetric dimethylarginine [ADMA], indoxyl sulfate [IS], p-cresyl sulfate [PCS], urea), cytokines (tumor necrosis factor alpha [TNF-α]), interleukins (interleukin-1 beta [IL-1β] and interleukin-6 [IL-6]), ions such as hydrogen, advanced glycation end products, and other metabolites (atrial natriuretic peptide [ANP]) ^[8,9]^. Research using adipocyte cell models and kidney disease rodent models showed that these compounds have profound consequences in the regulation of adipose tissue.

Adipose tissue is in a continuous state of dynamic remodeling depending on energy reserves and insulin sensitivity, and plays an important role in establishing homeostasis between the energy expenditure and inflammation as well as thermogenesis, a processes that can be significantly dysregulated in kidney disease. Also, adipose tissue produces multiple adipokines involved in appetite regulation, inflammation, and glucose metabolism and the regulation of these adipokines is significantly altered in patients with CKD ^[10]^. The metabolic changes that adipose tissue undergoes in kidney disease and the role that the adipose tissue plays in the co-morbidities of CKD patients will be examined in this article.

## 2. Adipose tissue lipid metabolism in kidney disease

The process of lipolysis results in triglyceride hydrolysis from lipid droplets for either energetic or signaling purposes ^[11]^. The main enzymes involved in lipolysis include adipose triglyceride lipase (ATGL), the rate-limiting enzyme, which hydrolyzes triglycerides into diglycerides; hormone-sensitive lipase (HSL), which degrades diglycerides into monoglycerides; and monoglyceride lipase, which breaks the last bond, generating the final free fatty acids (FFAs) and the glycerol backbone ^[12]^. Efficient ATGL enzyme activity requires binding to comparative gene identification 58 cofactor (CGI-58), which enhances its hydrolase activity ^[13]^. Lipolysis is a tightly regulated process in white adipose tissue (WAT), with low rates of triglyceride hydrolysis under basal conditions ^[11]^. Dysregulation of the basal lipolytic rate is present in several metabolic disorders ^[14]^ and is mediated by multiple factors, including endoplasmic reticulum (ER) stress, oxidative stress, and inflammation. Similarly, exposure to systemic uremic toxins promotes ER stress, oxidative stress, and inflammation in adipocytes, which leads to the changes in lipid mobilization ^[15–17]^.

Advanced CKD patients have smaller adipocyte size in WAT ^[18]^ and suffer from fat loss, which is associated with higher mortality risk ^[19]^. Furthermore, serum and plasma metabolic profiling of CKD patient cohorts have shown increased lipolysis markers and circulating FFAs ^[20–22]^. In end-stage renal disease (ESRD) patients on hemodialysis, high circulating FFAs levels are associated with increased mortality ^[23]^. In vitro studies have corroborated that adipocyte exposure to serum of CKD patients leads to a shift in lipid metabolism, with increased lipolysis and lipogenesis inhibition ^[24]^ in both human ^[25]^ and mouse ^[24,26]^ adipocytes. Dysregulation of lipid metabolism in adipocytes is also reproduced after exposure to specific uremic toxins such as IS and PCS ^[16,27,28]^. Similar to the human observations, animals that develop kidney disease develop fat loss and ectopic lipid redistribution ^[29]^. Administration of PCS to mice with normal kidney function was associated with loss of fat mass and lipid deposition in muscle and liver, similarly to mice that develop CKD ^[16]^.

### 2.1 CKD-induced ER and oxidative stress dysregulation of adipocyte lipolysis

The ER is a key cellular compartment that not only regulates membrane and secretory protein folding as well as protein post-translational modifications but also contributes to the regulation of lipolysis ^[30]^. Accumulation of unfolded proteins among other conditions can cause ER stress, leading to lipolysis ^[31,32]^ and, if unrestrained, ultimately to cell death ^[30,33]^. ER stress markers are increased in both ex vivo visceral WAT and primary adipocytes from 5/6 nephrectomy or subtotal nephrectomy rats, which have enhanced lipolysis due to increased activation of both HSL and ATGL. Moreover, 4-phenyl butyric acid, a chemical chaperone that reduces ER stress, alleviates lipolysis in a rat model of CKD by preventing ATGL activation by reducing its binding to CGI-58 cofactor ^[17]^. Finally, ER stress mediated by uremic toxins, such as ADMA, precedes and enhances adipocyte lipolysis by decreasing perilipin A ^[15]^, a key hormone for HSL translocation into the lipid droplet surface ^[12]^.

Under physiological conditions, reactive oxygen species (ROS) and reactive nitrogen species are produced as a byproduct of normal cellular metabolism ^[34]^. ROS are produced by NADPH oxidase (NOX) and dual oxidase enzymes in a regulated manner ^[35,36]^ and low level of ROS have a function in cellular processes such as regulation of intracellular signaling pathways ^[37]^ including adipocyte lipid metabolism ^[38]^. In excess, oxidative stress can lead to metabolic dysregulation or oxidation of proteins, lipids, and DNA, leading to cellular, tissue, and organ damage ^[34,39]^. In fact, CKD patients exhibit high levels of systemic ^[40–42]^ and tissue-specific oxidative stress, which includes WAT ^[18]^. This correlates with the accumulation of uremic toxins ^[43]^. Similarly, WAT of CKD animals have high oxidative stress ^[44]^, which promotes increased lipolysis as lipolysis can be abrogated by the action of the antioxidant *N*-acetyl cysteine in these models ^[45]^. Nonetheless, the mechanisms by which ROS causes adipocyte dysfunction in CKD are not well characterized. Uremic toxin accumulation, including IS ^[46]^, PCS ^[47]^, and urea ^[44]^, promotes ROS production in 3T3-L1 adipocytes. Some of the mechanisms involved in the increased cellular oxidative stress that results in lipid peroxidation and decreased lipogenesis by uremic toxins include upregulation of NOX isoform 4 ^[48]^, decreased antioxidant glutathione pools ^[47]^, and activation of sodium-potassium adenosine triphosphatase ^[28]^.

In summary, CKD results in adipocyte ER stress and increased WAT oxidative stress and both mechanisms contribute to heightened lipolysis, adipose tissue loss, increased FFA circulation, and ectopic lipid redistribution enhancing atherosclerosis and mortality in CKD.

## 3. WAT browning in kidney disease

Progressive adipose tissue loss is a pivotal component of metabolic disease-associated cachexia ^[49]^. WAT is specialized in lipid storage, and brown adipose tissue plays a role in thermogenesis. Beige adipocytes can also be found in WAT, arising through white adipocyte reprogramming by a process designated browning. Beige adipocytes are characterized by high uncoupling protein-1 expression and high mitochondrial activity ^[50,51]^. Increased browning of WAT could be beneficial in diseases such as obesity, but on the contrary, several recent studies have highlighted the detrimental effects of WAT browning in hypermetabolic diseases such as cancer and CKD ^[51,52]^. Studies in models of CKD have demonstrated that there is increased energy expenditure ^[53]^, a shift to multilocular fat cells ^[29]^ and mitochondrial enrichment ^[54]^ in WAT, which is consistent with increased adipose tissue browning in CKD. However, the mechanisms orchestrating WAT browning in CKD remain poorly understood. In a murine model of 5/6 or subtotal nephrectomy, Kir et al ^[55]^ showed that high levels of parathyroid hormone induced the expression of the thermogenic genes *Ucp1*, *Dio2*, *Cidea*, and *Pgc1a*. Similarly, high levels of ANP in CKD patients can induce browning in primary mouse adipocytes through an increase in expression of *Ucp1* and *Pgc1a* ^[56]^. Treatment with growth hormone ^[57]^, IL-1 receptor blockers such as anakinra ^[58]^ and Vitamin D supplementation ^[59]^ reduce WAT browning in CKD models highlighting several potential pathways that contribute to this process in CKD and that may be amenable to intervention. WAT browning is an underdeveloped area in CKD research but it may be an important mechanism of CKD-associated cachexia, which results in significant morbidity and mortality in ESRD patients.

## 4. Adipose and muscle tissue-mediated IR in kidney disease

Enhanced lipolysis in CKD leads to an increased release of FFAs ^[15,25,26]^, which will be distributed systemically to other organs promoting lipotoxicity and IR ^[60]^. Accordingly, CKD patients and animal models have IR ^[44,61,62]^ with hyperinsulinemia ^[63]^, which correlates with higher FFA levels and dyslipidemia ^[16,21,64]^. Uremic serum promotes human adipocyte lipolysis ^[25]^, and rodent models with CKD have ectopic lipid distribution ^[29]^ and suppressed IRS-1-associated phosphoinositide 3-kinase (PI3-K) activity in muscle ^[65]^, all processes that can result in the development of IR ^[66]^. Furthermore, uremic toxins such as PCS can modulate systemic IR by affecting the IRS/PI-3/AKT pathway through direct activation of ERK1/2 in rodent skeletal muscle ^[16]^. Alternatively, PCS ^[27]^ and carbamoyl-asparagine ^[67]^ can inhibit insulin-induced glucose uptake in rodent adipocytes. Furthermore, D’ Apolito et al showed that similar to rats with CKD, treatment of 3T3-L1 adipocytes with urea induced IR through increased mitochondrial ROS levels, leading to upregulation of resistin and retinol-binding protein 4, adipokines that result in IR. ROS promotes IRS-1 modification by *O*-GlcNAc, which attenuates its activity by decreasing tyrosine phosphorylation and consequently impairs insulin-stimulated glucose uptake ^[44]^ and glucose transporter type 4 (GLUT4) trafficking ^[68]^. Increased aldosterone levels in CKD patients also promote IR by a different mechanism ^[69]^. Hosoya et al ^[64]^ demonstrated that high aldosterone in WAT leads to mineralocorticoid receptor (MR) activation and consequent ADMA accumulation in adipocytes, which promotes oxidative stress. Increased oxidative stress leads to impairment of PI3-K activation and consequent insulin-mediated AKT phosphorylation, which can be salvaged by spironolactone, a MR blocker, in both CKD patients and rodents. Others also reported that metabolic acidosis ^[70]^ and angiotensin II ^[68]^ may play a role in the development of IR in CKD. Furthermore, IR leads to increased lipolysis, which in turn aggravates IR creating a vicious circle.

In summary, IR associated with CKD has multiple mechanisms affecting adipose and muscle tissue insulin and glucose pathways. Further studies are needed to determine their specific weights in patients with kidney disease in order to prioritize future therapeutic studies.

## 5. Kidney disease-induced adipose tissue inflammation

Chronic low-grade inflammation is common in patients with CKD, evident by their elevated peripheral levels of IL-6, TNF-α, interferon-gamma, IL-1β, and C-reactive protein (CRP). Inflammatory markers are correlated with increased levels of uremic toxins such as IS ^[42,43,71,72]^. Similarly, increased inflammation is present in a wide array of CKD rodent models ^[45,73,74]^. Inflammation is associated with poor outcomes in patients ^[75–77]^ and induces other co-morbidities in CKD such as atherosclerosis ^[78,79]^. The origins of inflammation in CKD are multifactorial. Similar to obesity ^[80]^, adipocyte dysregulation in CKD contributes to the increase in circulating inflammatory cytokines ^[81,82]^. Increased adipose tissue inflammation in CKD patients is independent of obesity status ^[26,45,83]^ but at the same time obesity-associated changes in fat mass in CKD patients also correlate with CRP levels and soluble CD163 levels, which is an activated macrophage marker ^[84]^. WAT contains not only adipocytes but also several other cell types, including immune cells. Although adipocytes are responsible for the secretion of several inflammatory cytokines, the non-adipocyte cells are the major contributors to the inflammatory cytokines attributed to WAT ^[45,85]^. The mechanisms driving CKD WAT inflammation are still unknown, but progress is being made to understand adipocyte and macrophage-derived inflammation.

Inflammatory cytokines are a major driver of adipose tissue metabolism and adipocytes themselves are a source of inflammatory cytokines ^[86]^. Adipocytes exposed to IS ^[46,48]^, PCS ^[28]^, ADMA ^[87]^, and advanced oxidation protein products ^[88]^ have increased expression and secretion of IL-6 and TNF-α. Similar to obesity where oxidative stress is a driver of increased WAT inflammation ^[89]^, NOX-mediated ROS production ^[46,88]^ leads to increased adipocyte inflammation through the NF-κB pathway in kidney disease ^[15]^. Furthermore, adipocytes exposed to macrophages that have been previously primed with serum from patients with CKD have increased inflammatory cytokine production, highlighting the close communication between adipocytes and stromal cells in CKD ^[26]^. Adipocyte production of inflammatory cytokines can in turn lead to IR ^[88]^ and promote lipolysis ^[15]^ via IL-6 ^[90]^ and TNF-α ^[87]^ within the adipose tissue as an autocrine effect.

Adipose tissue macrophages (ATMs) are another key player in metabolic disorders ^[91]^. Macrophage infiltration of adipose tissue is well described in obesity ^[92]^. Macrophages in adipose tissue are derived from migration of monocytes into WAT ^[92]^, self-renewal from tissue resident population ^[93]^, or chemokine ligand 2 (CCL2)-driven proliferation of resident macrophages ^[94]^. Recently, macrophage infiltration has been reported in both patient and animal models of CKD ^[18,45,48]^ being independent of body mass index (BMI) ^[26,83]^. WAT of CKD patients produce CCL2 ^[45,48]^, mainly by adipocytes ^[45]^, which leads to macrophage infiltration. Mechanisms involved in adipose tissue CCL2 production include NOX-mediated oxidative stress resulting from uremic toxin accumulation ^[15,28,48]^ and IL-6 ^[26]^.

Macrophages that migrated to obese WAT acquire a pro-inflammatory phenotype ^[95]^, and recent evidence suggests that CKD promotes macrophage activation ^[26,45]^. The mechanisms of ATM activation in CKD are still poorly understood. Uremic serum exposure or uremic toxin accumulation in in vitro macrophage cell models promotes a pro-inflammatory phenotype in a process mediated by both oxidative stress and NF-κB ^[26,96–98]^. Also, metabolite exposure, such as fatty acid palmitate, can lead to what is now termed macrophage metabolic activation, which results in an inflammatory phenotype similar to macrophage classical activation ^[99]^. Accordingly, macrophage/monocyte cell lines treated with serum of CKD patients have increased expression of the metabolic activation markers CD36, PLIN2, and ABCA1 and they also produce higher levels of inflammatory cytokines and CCL2 compared with exposure to serum from control patients ^[26]^. Furthermore, CKD potentiates a more robust inflammatory response to palmitate in peritoneal macrophages from 5/6 or subtotal nephrectomy rats than from controls ^[45]^.

In sum, adipocytes and macrophages within WAT are sources of inflammatory cytokines independent of adipose tissue mass and they show an inter-relationship. Studies on pro-inflammatory activation of macrophages by metabolic products have been previously described but understudied in CKD and this area of research may identify one of the key sites of the heightened inflammatory state of CKD patients.

## 6. Adipokine dysregulation in CKD

WAT produce a variety of hormones and cytokines termed adipokines. A summary of adipokines dysregulated in uremia is shown in Table 1. We will review with some detail adipokines that have been more widely studied below.

**Table 1.** Summary of the main adipokines dysregulated in CKD.

Adipokine	Effects in renal disease
Adiponectin	•-Increased circulating adiponectin levels ^[100–103]^ •-Increased adiponectin production from adipose tissue ^[71]^ •-Increased adiponectin receptor (R1) expression ^[104]^ •-Increased adiponectin resistance at a post receptor level ^[104]^
Leptin	•-Rise in circulating leptin levels ^[105–108]^ and decrease in sOBR/SL leading to appetite suppression, increased energy expenditure ^[109]^ •-Increased inflammation ^[110,111]^ •-Decreased neutrophil function ^[112]^
Ghrelin (acyl, des-acyl, and obestatin)	•-Increased circulating levels of anorexigenic forms ^[113–115]^
Resistin	•-Increased level of circulating resistin leading to increased inflammatory markers such as TNF-α and CRP ^[44]^
RBP4	•-Promotes IR in adipocytes ^[44]^
Pro-inflammatory cytokines (Il-1; IL-6; TNF-α)	•-Increased circulating levels in CKD contributing to: -Low-grade chronic inflammation ^[81,82]^ -Increased adipocyte lipolysis ^[15,87,90]^ -IR ^[88]^ -Anorexia and PEW ^[116]^ •-Associated with worse patient outcomes ^[75,77]^
CCL2	•-Promotes macrophage infiltration in WAT ^[45,48]^

CCL2, chemokine ligand 2; CKD, chronic kidney disease; CRP, C-reactive protein; IL-6, interleukin-6; IR, insulin resistance; PEW, protein energy wasting; RBP4, retinol binding protein 4; SL, serum leptin; sOBR, soluble leptin-binding receptor; TNF-α, tumor necrosis factor alpha; WAT, white adipose tissue.

### 6.1 Adiponectin

Adiponectin is a 30 kDa adipokine that belongs to the family of C1q/TNF-related proteins. It is considered an anti-inflammatory, anti-diabetic, and anti-atherogenic cytokine and is found in relatively high plasma levels (2–20 µg/mL). Serum adiponectin levels decrease with increase in fat mass and in IR ^[117]^. Adiponectin circulates in three different isoforms including low-molecular weight trimers, medium-molecular weight hexamer, high-molecular weight oligomers and a globular monomer ^[117]^. Adiponectin binds to three different adiponectin receptors (AdipoR1, R2, and T-cadherin). AdipoR1 and AdipoR2 are G-protein coupled receptors that are expressed ubiquitously ^[118,119]^. AdipoR1 receptor activates the adenosine monophosphate-activated protein kinase (AMPK) signaling pathway preferentially in muscle and results in glucose uptake via GLUT4. AdipoR2 activates peroxisome proliferator-activated receptor-alpha signaling predominantly in the liver promoting fatty acid oxidation and energy expenditure reducing oxidative stress and inflammation ^[118,120]^. Together AdipoR1 and Adipo R2 stimulate glucose transport and fatty acid oxidation resulting in decreased triglyceride content and increase in insulin sensitivity. Given its size and molecular weight, adiponectin multimers are not excreted by the kidney, but adiponectin monomers and the degradation products can be excreted in urine ^[121]^.

Adiponectin is minimally excreted in the kidneys in the absence of kidney disease ^[121]^. CKD patients have elevated levels of serum adiponectin and their levels correlate with progression of kidney damage ^[100]^. Moreover, serum adiponectin levels are elevated in patients with albuminuria and correlate positively with the degree of urine albumin/creatinine ratio ^[102]^. Patients with albuminuria due to IgA nephropathy and diabetic nephropathy demonstrated increased urinary excretion of adiponectin, suggesting also an enhanced filtration of circulating adiponectin with albumin ^[121]^. ESRD patients are found to have the highest levels of serum adiponectin and their levels do not differ between patients on hemodialysis or peritoneal dialysis ^[101,103]^. ESRD patients have increased production of adiponectin in subcutaneous and visceral adipose tissue with upregulation of adiponectin mRNA levels ^[71]^. In addition to elevated adiponectin levels, adiponectin receptor is also upregulated in muscle, adipose tissue, and peripheral blood mononuclear cells in ESRD patients. Despite increased adiponectin and adiponectin receptor in CKD, our laboratory has demonstrated adiponectin post receptor resistance in muscle tissue of kidney disease patients with decreased levels of pACC and CPT-1 levels despite higher AdipoR1 and pAMPK expression ^[104]^. Adiponectin resistance in skeletal muscle could contribute to the state of IR in patients with CKD. Although there has been evidence of adiponectin resistance in CKD, the etiology for this post receptor resistance and its effects on metabolism remain unknown.

### 6.2 Leptin

Leptin is a satiety hormone produced by adipocytes that acts on various central and peripheral receptors to regulate appetite. It is responsible for the development of a feeding pattern in an individual. Leptin level is directly associated with body fat mass and communicates the state of energy levels to the brain to regulate appetite and body mass. Leptin levels are not only naturally elevated in obesity but also genetic ablation of leptin gene can induce obesity ^[122]^.

Leptin acts on the hypothalamic melanocortin system by decreasing the expression of neuropeptide Y (NPY), an appetite stimulator, and increasing the levels of anorexigenic neuropeptides such as proopiomelanocortin (POMC) ^[123]^. Activation of POMC neurons release alpha-melanocyte-stimulating hormone (α-MSH) that activates the type 4 melanocortin receptor-4 (MC4R), leading to suppressed appetite and increased energy expenditure ^[105,124,125]^.

There are several isoforms of leptin receptors (OBR a–e). OBRb is expressed in hypothalamus and regulates appetite and satiety. OBRe is a circulating soluble leptin-binding receptor (sOBR) without membrane anchor function that can be measured in blood as an indicator of leptin activity. OBRe competitively binds leptin making it unavailable for biological activity. Therefore, elevated sOBR/serum leptin (SL) ratio is an indicator of leptin resistance, which can be seen in obesity ^[109]^.

CKD patients have elevated leptin levels after adjusting for their BMI ^[105–108]^ and dialysis patients demonstrate about four-fold rise in the leptin levels ^[108]^. Leptin is produced by adipocytes and is filtered in the glomeruli. After filtration, leptin is further degraded by megalin in the renal tubules before its excretion in the urine ^[126]^. Therefore, urine contains negligible quantity of leptin. The origin of hyperleptinemia in CKD patients is most likely multifactorial and not only involves impaired renal elimination but also increased production by adipocytes ^[127]^. Despite a study showing downregulation of *lep* gene in the adipocytes of patients with CKD, in vitro studies have demonstrated that adipocytes exposed to uremic serum and inflammatory markers such as TNF-α and IL-6 have increased synthesis and secretion of leptin ^[105]^. Due to its role in appetite regulation and as energy expenditure, leptin plays a role in protein energy wasting (PEW) in CKD patients and this will be discussed in the next section. Additionally, in vitro studies have shown that hyperleptinemia alters neutrophil function in dialysis patients with decreased oxidative burst and reduced chemotaxis increasing infection risk ^[112]^.

### 6.3 Adipokines and PEW in CKD patients

PEW is a common complication of CKD with a prevalence of 30%–60% in patients with ESRD and is associated with increased mortality. PEW is also termed as uremic cachexia and its pathophysiology is similar to that of HIV and cancer. PEW manifests as anorexia, increased energy expenditure, and enhanced lipolysis ^[116]^. Dysregulation of the hypothalamic melanocortin system (the appetite center) by various adipokines and cytokines including leptin plays the key role in PEW ^[116]^.

Despite the rise in leptin levels seen in CKD patients, sOBR levels remain normal ^[109]^, resulting in a low sOBR/SL ratio. Low sOBR/SL ratio will increase free leptin circulation causing appetite suppression and energy expenditure. Moreover, because CKD patients with elevated IL-6 had a significantly lower sOBR/SL ratio, this could reflect another mechanism by which inflammation via leptin worsens PEW in CKD ^[109]^.

Agouti-related peptide (AgRP) is also an appetite stimulator ^[123]^ and activation of NPY/AgRP by ghrelin results in suppression of melanocortin producing neurons to stimulate eating ^[116]^. Other adipokines including leptin, adiponectin and resistin, and cytokines such as IL-1β, IL-6, and TNF-α suppress the NPY/AgRP and activates the POMC/α-MSH resulting in anorexia and increased energy expenditure ^[105,116]^. Animal models have demonstrated leptin-mediated uremic cachexia and reversal of symptoms by intrathecal injection of AgRP ^[128,129]^. A similar study in cancer cachexia animal models has demonstrated reversal of cachexia with a peripherally administered MC4-R antagonist ^[130]^. These studies highlight potential new avenues for therapeutics involving adipose tissue-regulated appetite modulators.

## 7. Adipose tissue dysfunction and CKD progression

Altered WAT metabolism and function are direct consequences of kidney disease. Furthermore, WAT dysfunction due to metabolic disorders has a role in CKD development, progression, and co-morbidities. Specifically, the role of obesity-related adipose tissue dysfunction in the promotion and maintenance of kidney disease has been extensively reviewed and is outside of the scope of this mini-review ^[131–133]^. Adipose tissue dysfunction in advanced kidney disease and obesity share similarities as most of the key cytokines, metabolites, and hormones that are dysregulated in uremia have been demonstrated to cause kidney injury in obesity models as well. For instance, altered lipid metabolism and ectopic lipid accumulation in obesity affect renal tubular cells leading to proximal tubule injury ^[134,135]^. Also, obesity-driven adipose tissue inflammation contributes to systemic chronic inflammation and induces abnormal kidney function ^[136]^. Finally, adipokine dysregulation in obesity such as hyperleptinemia ^[137–139]^ and hypo-adiponectinemia ^[140,141]^ promote CKD by increasing kidney fibrosis and inflammation. Hence, it is likely that the adipose tissue dysfunction in uremia promotes progression of CKD in a feed forward loop. Despite the clear evidence of bidirectional crosstalk between WAT and the kidney, there are significant gaps in the understanding of how adipose tissue heightens kidney disease progression outside of the context of excess adiposity and will require further research.

## 8. Conclusion/future perspectives

**Figure 1.**
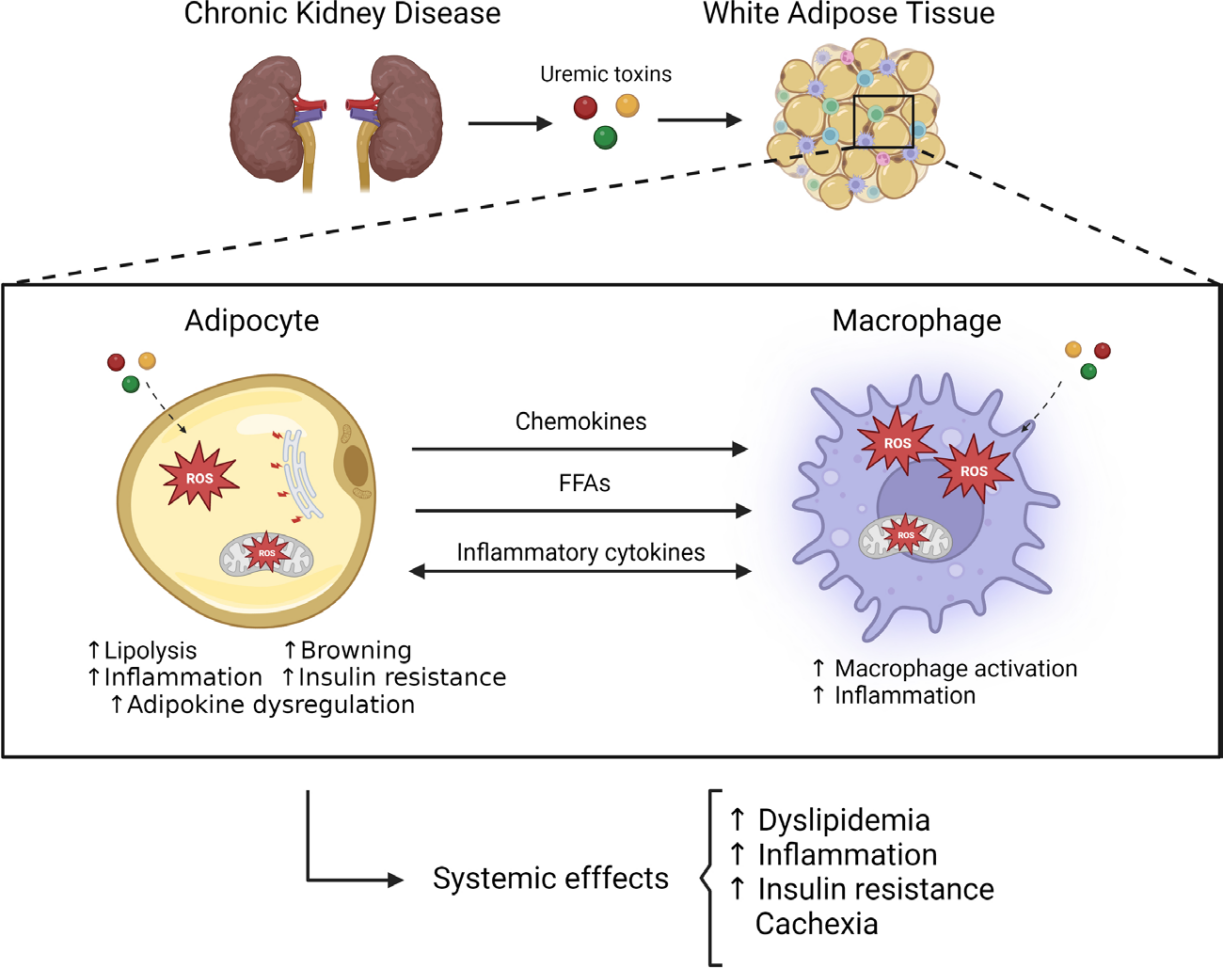
**Mechanisms of adipose tissue dysfunction in CKD**. Kidney disease results in the accumulation of different metabolic waste products or uremic toxins. These uremic toxins can affect both adipocytes and macrophages and promote adipose tissue dysfunction. In macrophages in adipose tissue, uremic toxins promote excess ROS leading to an inflammatory phenotype. Also, exposure to uremic toxins in adipocytes results in ER stress as well as ROS, which facilitate lipolysis, browning and adipokine dysregulation leading to insulin resistance and inflammation. There is crosstalk between adipocytes and macrophages with adipocyte cytokines facilitating macrophage recruitment to adipose tissue and FFA from lipolysis promoting macrophage metabolic activation with increased inflammatory cytokine production. In addition, macrophages exposed to uremic serum promote inflammatory adipokine production. In sum, adipose tissue metabolic dysregulation in CKD results in increased inflammatory cytokine production, dyslipemia, and insulin resistance, which promote atherosclerosis, as well as increased thermogenesis and adipocyte catabolism, which are both associated with cachexia. CKD, chronic kidney disease; ER, endoplasmic reticulum; FFA, free fatty acids; ROS, reactive oxygen species.

WAT involvement in metabolic disorders such as obesity and diabetes is a highly researched topic but studies on the mechanisms by which CKD disrupt WAT function are still scarce. Adipose tissue metabolism is significantly altered by kidney disease and multiple mechanisms are involved (Figure 1). Some of these mechanisms share similarities with obesity and diabetes, although there are unique features of CKD as well. Adipose tissue dysregulation contributes to many of the detrimental effects of CKD including inflammation, atherosclerosis, IR, and cachexia. In depth research is needed to understand drivers of adipose tissue dysfunction in CKD and should include studies on the microenvironment of WAT and its impact on inflammation, adipokine, and adipose tissue thermogenesis contribution to energy wasting and cachexia as well as adipose tissue catabolism and its role in IR and atherosclerosis. Mechanistic research on the effects of CKD on WAT could reveal new biomarkers and targets for pharmacologic interventions, which could have clinical practice implications in the treatment of CKD and its related co-morbidities.

## Author contributions

E.S. and P.S. wrote the article. M.P.M. wrote the article and had primary responsibility for final content. All authors have read and approved the final article.

## Conflicts of interest

The authors declare they have no conflicts of interest.

## Funding

The work was primarily funded by the National Institute of Diabetes and Digestive and Kidney Disease (NIDDK) Division of Intramural Research projects R01DK111574 (to M.P.M.).

## Acknowledgments

The article figure was created with BioRender.com.

## References

[1] Centers for Disease Control and Prevention. Chronic Kidney Disease in the United States, 2021. Available from: https://www.cdc.gov/kidneydisease/publications-resources/ckd-national-facts.html#:~:text=CKD%20is%20more%20common%20in,Hispanic%20Asian%20adults%20(13%25). [Acessed April 3, 2023].

[2] Coresh J. Update on the burden of CKD. J Am Soc Nephrol. 2017;28(4):1020–2. 28302756 10.1681/ASN.2016121374 PMC5373470

[3] Pecoits-Filho R Lindholm B Stenvinkel P. The malnutrition, inflammation, and atherosclerosis (MIA) syndrome—the heart of the matter. Nephrol Dial Transplant. 2002;17(Suppl 11):28–31. 10.1093/ndt/17.suppl_11.28 12386254

[4] Stenvinkel P Alvestrand A. Inflammation in end-stage renal disease: sources, consequences, and therapy. Semin Dial. 2002;15(5):329–37. 12358637 10.1046/j.1525-139x.2002.00083.x

[5] Go AS Chertow GM Fan D. Chronic kidney disease and the risks of death, cardiovascular events, and hospitalization. N Engl J Med. 2004;351(13):1296–305. 15385656 10.1056/NEJMoa041031

[6] Silverstein DM. Inflammation in chronic kidney disease: role in the progression of renal and cardiovascular disease. Pediatr Nephrol. 2009;24(8):1445–52. 19083024 10.1007/s00467-008-1046-0

[7] Leyking S Fliser D. Insulin resistance in CKD. Clin J Am Soc Nephrol. 2014;9(4):638–40. 24677558 10.2215/CJN.01290214 PMC3974344

[8] Vanholder R De Smet R Glorieux G; European Uremic Toxin Work Group (EUTox). Review on uremic toxins: classification, concentration, and interindividual variability. Kidney Int. 2003;63(5):1934–43. 12675874 10.1046/j.1523-1755.2003.00924.x

[9] Rosner MH Reis T Husain-Syed F. Classification of uremic toxins and their role in kidney failure. Clin J Am Soc Nephrol. 2021;16(12):1918–28. 34233920 10.2215/CJN.02660221 PMC8729494

[10] Lee MJ Wu Y Fried SK. Adipose tissue remodeling in pathophysiology of obesity. Curr Opin Clin Nutr Metab Care. 2010;13(4):371–6. 20531178 10.1097/MCO.0b013e32833aabef PMC3235038

[11] Li Y Li Z Ngandiri DA. The molecular brakes of adipose tissue lipolysis. Front Physiol. 2022;13:826314. 35283787 10.3389/fphys.2022.826314 PMC8907745

[12] Duncan RE Ahmadian M Jaworski K. Regulation of lipolysis in adipocytes. Annu Rev Nutr. 2007;27:79–101. 17313320 10.1146/annurev.nutr.27.061406.093734 PMC2885771

[13] Lass A Zimmermann R Haemmerle G. Adipose triglyceride lipase-mediated lipolysis of cellular fat stores is activated by CGI-58 and defective in Chanarin-Dorfman Syndrome. Cell Metab. 2006;3(5):309–19. 16679289 10.1016/j.cmet.2006.03.005

[14] Nielsen TS Jessen N Jorgensen JO. Dissecting adipose tissue lipolysis: molecular regulation and implications for metabolic disease. J Mol Endocrinol. 2014;52(3):R199–222. 24577718 10.1530/JME-13-0277

[15] Zhou QG Zhou M Hou FF. Asymmetrical dimethylarginine triggers lipolysis and inflammatory response via induction of endoplasmic reticulum stress in cultured adipocytes. Am J Physiol Endocrinol Metab. 2009;296(4):E869–78. 19208851 10.1152/ajpendo.91011.2008

[16] Koppe L Pillon NJ Vella RE. p-Cresyl sulfate promotes insulin resistance associated with CKD. J Am Soc Nephrol. 2013;24(1):88–99. 23274953 10.1681/ASN.2012050503 PMC3537215

[17] Zhu Y Chen YL Li C. The effect of inhibition of endoplasmic reticulum stress on lipolysis in white adipose tissue in a rat model of chronic kidney disease. Acta Pharmacol Sin. 2014;35(3):356–62. 24442147 10.1038/aps.2013.177 PMC4647896

[18] Gertow J Ng CZ Mamede Branca RM. Altered protein composition of subcutaneous adipose tissue in chronic kidney disease. Kidney Int Rep. 2017;2(6):1208–18. 29270529 10.1016/j.ekir.2017.07.007 PMC5733748

[19] Kalantar-Zadeh K Kuwae N Wu DY. Associations of body fat and its changes over time with quality of life and prospective mortality in hemodialysis patients. Am J Clin Nutr. 2006;83(2):202–10. 16469976 10.1093/ajcn/83.2.202

[20] Rhee EP Souza A Farrell L. Metabolite profiling identifies markers of uremia. J Am Soc Nephrol. 2010;21(6):1041–51. 20378825 10.1681/ASN.2009111132 PMC2900954

[21] Chen H Chen L Liu D. Combined clinical phenotype and lipidomic analysis reveals the impact of chronic kidney disease on lipid metabolism. J Proteome Res. 2017;16(4):1566–78. 28286957 10.1021/acs.jproteome.6b00956

[22] Baek J He C Afshinnia F. Lipidomic approaches to dissect dysregulated lipid metabolism in kidney disease. Nat Rev Nephrol. 2022;18(1):38–55. 34616096 10.1038/s41581-021-00488-2 PMC9146017

[23] Sikorska-Wisniewska M Mika A Sledzinski T. Associations between serum saturated fatty acids content and mortality in dialysis patients. J Clin Med. 2022;11(17):5051. 36078983 10.3390/jcm11175051 PMC9457217

[24] Pelletier CC Koppe L Croze ML. White adipose tissue overproduces the lipid-mobilizing factor zinc alpha2-glycoprotein in chronic kidney disease. Kidney Int. 2013;83(5):878–86. 23423258 10.1038/ki.2013.9 PMC3938447

[25] Axelsson J Astrom G Sjolin E. Uraemic sera stimulate lipolysis in human adipocytes: role of perilipin. Nephrol Dial Transplant. 2011;26(8):2485–91. 21220757 10.1093/ndt/gfq755

[26] Martos-Rus C Katz-Greenberg G Lin Z. Macrophage and adipocyte interaction as a source of inflammation in kidney disease. Sci Rep. 2021;11(1):2974. 33536542 10.1038/s41598-021-82685-4 PMC7859223

[27] Tanaka S Yano S Sheikh AM. Effects of uremic toxin p-cresol on proliferation, apoptosis, differentiation, and glucose uptake in 3T3-L1 cells. Artif Organs. 2014;38(7):566–71. 24417700 10.1111/aor.12252

[28] Bartlett DE Miller RB Thiesfeldt S. Uremic toxins activates Na/K-ATPase oxidant amplification loop causing phenotypic changes in adipocytes in in vitro models. Int J Mol Sci . 2018;19(9):2685. 30201874 10.3390/ijms19092685 PMC6164729

[29] Zhao HL Sui Y Guan J. Fat redistribution and adipocyte transformation in uninephrectomized rats. Kidney Int. 2008;74(4):467–77. 18496513 10.1038/ki.2008.195

[30] Schroder M Kaufman RJ. ER stress and the unfolded protein response. Mutat Res. 2005;569(1-2):29–63. 15603751 10.1016/j.mrfmmm.2004.06.056

[31] Deng J Liu S Zou L. Lipolysis response to endoplasmic reticulum stress in adipose cells. J Biol Chem. 2012;287(9):6240–9. 22223650 10.1074/jbc.M111.299115 PMC3307255

[32] Bogdanovic E Kraus N Patsouris D. Endoplasmic reticulum stress in adipose tissue augments lipolysis. J Cell Mol Med. 2015;19(1):82–91. 25381905 10.1111/jcmm.12384 PMC4288352

[33] Xu C Bailly-Maitre B Reed JC. Endoplasmic reticulum stress: cell life and death decisions. J Clin Invest. 2005;115(10):2656–64. 16200199 10.1172/JCI26373 PMC1236697

[34] Di Meo S Reed TT Venditti P. Role of ROS and RNS sources in physiological and pathological conditions. Oxid Med Cell Longev. 2016;2016:1245049. 27478531 10.1155/2016/1245049 PMC4960346

[35] Lambeth JD. NOX enzymes and the biology of reactive oxygen. Nat Rev Immunol. 2004;4(3):181–9. 15039755 10.1038/nri1312

[36] Vermot A Petit-Hartlein I Smith SME. NADPH Oxidases (NOX): an overview from discovery, molecular mechanisms to physiology and pathology. Antioxidants (Basel). 2021;10(6):890. 34205998 10.3390/antiox10060890 PMC8228183

[37] Daenen K Andries A Mekahli D. Oxidative stress in chronic kidney disease. Pediatr Nephrol. 2019;34(6):975–91. 30105414 10.1007/s00467-018-4005-4

[38] Abou-Rjeileh U Contreras GA. Redox regulation of lipid mobilization in adipose tissues. Antioxidants (Basel). 2021;10(7):1090. 10.3390/antiox10071090 PMC8301038 34356323

[39] Rapa SF Di Iorio BR Campiglia P. Inflammation and oxidative stress in chronic kidney disease-potential therapeutic role of minerals, vitamins and plant-derived metabolites. Int J Mol Sci . 2019;21(1):263. 31906008 10.3390/ijms21010263 PMC6981831

[40] Witko-Sarsat V Friedlander M Capeillere-Blandin C. Advanced oxidation protein products as a novel marker of oxidative stress in uremia. Kidney Int. 1996;49(5):1304–13. 8731095 10.1038/ki.1996.186

[41] Handelman GJ Walter MF Adhikarla R. Elevated plasma F2-isoprostanes in patients on long-term hemodialysis. Kidney Int. 2001;59(5):1960–6. 11318969 10.1046/j.1523-1755.2001.0590051960.x

[42] Oberg BP McMenamin E Lucas FL. Increased prevalence of oxidant stress and inflammation in patients with moderate to severe chronic kidney disease. Kidney Int. 2004;65(3):1009–16. 14871421 10.1111/j.1523-1755.2004.00465.x

[43] Rossi M Campbell KL Johnson DW. Protein-bound uremic toxins, inflammation and oxidative stress: a cross-sectional study in stage 3-4 chronic kidney disease. Arch Med Res. 2014;45(4):309–17. 24751327 10.1016/j.arcmed.2014.04.002

[44] D’Apolito M Du X Zong H. Urea-induced ROS generation causes insulin resistance in mice with chronic renal failure. J Clin Invest. 2010;120(1):203–13. 19955654 10.1172/JCI37672 PMC2798674

[45] Xiang DM Song XZ Zhou ZM. Chronic kidney disease promotes chronic inflammation in visceral white adipose tissue. Am J Physiol Renal Physiol. 2017;312(4):F689–701. 28100503 10.1152/ajprenal.00584.2016

[46] Stockler-Pinto MB Saldanha JF Yi D. The uremic toxin indoxyl sulfate exacerbates reactive oxygen species production and inflammation in 3T3-L1 adipose cells. Free Radic Res. 2016;50(3):337–44. 26617268 10.3109/10715762.2015.1125996

[47] Koppe L Croze ML Monteiro EB. The protein-bound uremic toxin p-cresyl-sulfate promotes intracellular ROS production and lipid peroxidation in 3T3-L1 adipose cells. Biochimie. 2021;189:137–43. 34217821 10.1016/j.biochi.2021.06.020

[48] Tanaka S Watanabe H Nakano T. Indoxyl sulfate contributes to adipose tissue inflammation through the activation of NADPH oxidase. Toxins (Basel). 2020;12(8):502. 32764271 10.3390/toxins12080502 PMC7472142

[49] Abdullahi A Jeschke MG. White adipose tissue browning: a double-edged sword. Trends Endocrinol Metab. 2016;27(8):542–52. 27397607 10.1016/j.tem.2016.06.006 PMC5234861

[50] Wu J Bostrom P Sparks LM. Beige adipocytes are a distinct type of thermogenic fat cell in mouse and human. Cell. 2012;150(2):366–76. 22796012 10.1016/j.cell.2012.05.016 PMC3402601

[51] Petruzzelli M Schweiger M Schreiber R. A switch from white to brown fat increases energy expenditure in cancer-associated cachexia. Cell Metab. 2014;20(3):433–47. 25043816 10.1016/j.cmet.2014.06.011

[52] Cheung WW Cherqui S Ding W. Muscle wasting and adipose tissue browning in infantile nephropathic cystinosis. J Cachexia Sarcopenia Muscle. 2016;7(2):152–64. 27493869 10.1002/jcsm.12056 PMC4864942

[53] Neyra R Chen KY Sun M. Increased resting energy expenditure in patients with end-stage renal disease. JPEN J Parenter Enteral Nutr. 2003;27(1):36–42. 12549596 10.1177/014860710302700136

[54] Zhao HL Sui Y He L. Lipid partitioning after uninephrectomy. Acta Diabetol. 2011;48(4):317–28. 21528432 10.1007/s00592-011-0286-9

[55] Kir S Komaba H Garcia AP. PTH/PTHrP receptor mediates cachexia in models of kidney failure and cancer. Cell Metab. 2016;23(2):315–23. 26669699 10.1016/j.cmet.2015.11.003 PMC4749423

[56] Luce M Barba C Yi D. Accumulation of natriuretic peptides is associated with protein energy wasting and activation of browning in white adipose tissue in chronic kidney disease. Kidney Int. 2020;98(3):663–72. 32739210 10.1016/j.kint.2020.03.027

[57] Mak RH Gunta S Oliveira EA. Growth hormone improves adipose tissue browning and muscle wasting in mice with chronic kidney disease-associated cachexia. Int J Mol Sci . 2022;23(23):15310. 36499637 10.3390/ijms232315310 PMC9740214

[58] Cheung WW Zheng R Hao S. The role of IL-1 in adipose browning and muscle wasting in CKD-associated cachexia. Sci Rep. 2021;11(1):15141. 34302016 10.1038/s41598-021-94565-y PMC8302616

[59] Cheung WW Ding W Hoffman HM. Vitamin D ameliorates adipose browning in chronic kidney disease cachexia. Sci Rep. 2020;10(1):14175. 32843714 10.1038/s41598-020-70190-z PMC7447759

[60] Sears B Perry M. The role of fatty acids in insulin resistance. Lipids Health Dis. 2015;14:121. 26415887 10.1186/s12944-015-0123-1 PMC4587882

[61] DeFronzo RA Alvestrand A Smith D. Insulin resistance in uremia. J Clin Invest. 1981;67(2):563–8. 7007440 10.1172/JCI110067 PMC370600

[62] Bodlaj G Berg J Pichler R. Prevalence, severity and predictors of HOMA-estimated insulin resistance in diabetic and nondiabetic patients with end-stage renal disease. J Nephrol. 2006;19(5):607–12. 17136689

[63] Feneberg R Sparber M Veldhuis JD. Altered temporal organization of plasma insulin oscillations in chronic renal failure. J Clin Endocrinol Metab. 2002;87(5):1965–73. 11994326 10.1210/jcem.87.5.8453

[64] Hosoya K Minakuchi H Wakino S. Insulin resistance in chronic kidney disease is ameliorated by spironolactone in rats and humans. Kidney Int. 2015;87(4):749–60. 25337775 10.1038/ki.2014.348

[65] Bailey JL Zheng B Hu Z. Chronic kidney disease causes defects in signaling through the insulin receptor substrate/phosphatidylinositol 3-kinase/Akt pathway: implications for muscle atrophy. J Am Soc Nephrol. 2006;17(5):1388–94. 16611720 10.1681/ASN.2004100842

[66] Saltiel AR. Insulin signaling in health and disease. J Clin Invest. 2021;131(1):e142241. 33393497 10.1172/JCI142241 PMC7773347

[67] Kraus LM Traxinger R Kraus AP. Uremia and insulin resistance: N-carbamoyl-asparagine decreases insulin-sensitive glucose uptake in rat adipocytes. Kidney Int. 2004;65(3):881–7. 14871407 10.1111/j.1523-1755.2004.00456.x

[68] Cao W Shi M Wu L. A renal-cerebral-peripheral sympathetic reflex mediates insulin resistance in chronic kidney disease. EBioMedicine. 2018;37:281–93. 30429087 10.1016/j.ebiom.2018.10.054 PMC6286258

[69] Quinkler M Zehnder D Eardley KS. Increased expression of mineralocorticoid effector mechanisms in kidney biopsies of patients with heavy proteinuria. Circulation. 2005;112(10):1435–43. 16145013 10.1161/CIRCULATIONAHA.105.539122

[70] Reaich D Graham KA Channon SM. Insulin-mediated changes in PD and glucose uptake after correction of acidosis in humans with CRF. Am J Physiol. 1995;268(1 Pt 1):E121–6. 7840169 10.1152/ajpendo.1995.268.1.E121

[71] Martinez Cantarin MP Waldman SA Doria C. The adipose tissue production of adiponectin is increased in end-stage renal disease. Kidney Int. 2013;83(3):487–94. 23283133 10.1038/ki.2012.421 PMC3587362

[72] Gupta J Mitra N Kanetsky PA; CRIC Study Investigators. Association between albuminuria, kidney function, and inflammatory biomarker profile in CKD in CRIC. Clin J Am Soc Nephrol. 2012;7(12):1938–46. 23024164 10.2215/CJN.03500412 PMC3513744

[73] Guo X Xu L Velazquez H. Kidney-targeted renalase agonist prevents cisplatin-induced chronic kidney disease by inhibiting regulated necrosis and inflammation. J Am Soc Nephrol. 2022;33(2):342–56. 34921111 10.1681/ASN.2021040439 PMC8819981

[74] Junho CVC Gonzalez-Lafuente L Neres-Santos RS. Klotho relieves inflammation and exerts a cardioprotective effect during renal ischemia/reperfusion-induced cardiorenal syndrome. Biomed Pharmacother. 2022;153:113515. 36068956 10.1016/j.biopha.2022.113515

[75] Stenvinkel P Heimburger O Paultre F. Strong association between malnutrition, inflammation, and atherosclerosis in chronic renal failure. Kidney Int. 1999;55(5):1899–911. 10231453 10.1046/j.1523-1755.1999.00422.x

[76] Wang AY Woo J Lam CW. Is a single time point C-reactive protein predictive of outcome in peritoneal dialysis patients? J Am Soc Nephrol. 2003;14(7):1871–9. 12819248 10.1097/01.asn.0000070071.57901.b3

[77] Cohen SD Phillips TM Khetpal P. Cytokine patterns and survival in haemodialysis patients. Nephrol Dial Transplant. 2010;25(4):1239–43. 20007982 10.1093/ndt/gfp625

[78] Honda H Qureshi AR Axelsson J. Obese sarcopenia in patients with end-stage renal disease is associated with inflammation and increased mortality. Am J Clin Nutr. 2007;86(3):633–8. 17823427 10.1093/ajcn/86.3.633

[79] Carrero JJ Stenvinkel P. Persistent inflammation as a catalyst for other risk factors in chronic kidney disease: a hypothesis proposal. Clin J Am Soc Nephrol. 2009;4(Suppl 1):S49–55. 19996005 10.2215/CJN.02720409

[80] Russo L Lumeng CN. Properties and functions of adipose tissue macrophages in obesity. Immunology. 2018;155(4):407–17. 30229891 10.1111/imm.13002 PMC6230999

[81] Zoccali C Mallamaci F Tripepi G. Adipose tissue as a source of inflammatory cytokines in health and disease: focus on end-stage renal disease. Kidney Int Suppl. 2003;63(84):S65–8. 12694312 10.1046/j.1523-1755.63.s84.50.x

[82] Axelsson J Rashid Qureshi A Suliman ME. Truncal fat mass as a contributor to inflammation in end-stage renal disease. Am J Clin Nutr. 2004;80(5):1222–9. 15531669 10.1093/ajcn/80.5.1222

[83] Martinez Cantarin MP Whitaker-Menezes D Lin Z. Uremia induces adipose tissue inflammation and muscle mitochondrial dysfunction. Nephrol Dial Transplant. 2017;32(6):943–51. 28605780 10.1093/ndt/gfx050

[84] Axelsson J Moller HJ Witasp A. Changes in fat mass correlate with changes in soluble sCD163, a marker of mature macrophages, in patients with CKD. Am J Kidney Dis. 2006;48(6):916–25. 17162146 10.1053/j.ajkd.2006.08.022

[85] Kershaw EE Flier JS. Adipose tissue as an endocrine organ. J Clin Endocrinol Metab. 2004;89(6):2548–56. 15181022 10.1210/jc.2004-0395

[86] Coppack SW. Pro-inflammatory cytokines and adipose tissue. Proc Nutr Soc. 2001;60(3):349–56. 11681809 10.1079/pns2001110

[87] Zhang HH Halbleib M Ahmad F. Tumor necrosis factor-alpha stimulates lipolysis in differentiated human adipocytes through activation of extracellular signal-related kinase and elevation of intracellular cAMP. Diabetes. 2002;51(10):2929–35. 12351429 10.2337/diabetes.51.10.2929

[88] Zhou QG Zhou M Lou AJ. Advanced oxidation protein products induce inflammatory response and insulin resistance in cultured adipocytes via induction of endoplasmic reticulum stress. Cell Physiol Biochem. 2010;26(4-5):775–86. 21063115 10.1159/000322345

[89] Furukawa S Fujita T Shimabukuro M. Increased oxidative stress in obesity and its impact on metabolic syndrome. J Clin Invest. 2004;114(12):1752–61. 15599400 10.1172/JCI21625 PMC535065

[90] Trujillo ME Sullivan S Harten I. Interleukin-6 regulates human adipose tissue lipid metabolism and leptin production in vitro. J Clin Endocrinol Metab. 2004;89(11):5577–82. 15531514 10.1210/jc.2004-0603

[91] Ruggiero AD Key CC Kavanagh K. Adipose tissue macrophage polarization in healthy and unhealthy obesity. Front Nutr. 2021;8:625331. 33681276 10.3389/fnut.2021.625331 PMC7925825

[92] Yu R Kim CS Kwon BS. Mesenteric adipose tissue-derived monocyte chemoattractant protein-1 plays a crucial role in adipose tissue macrophage migration and activation in obese mice. Obesity (Silver Spring). 2006;14(8):1353–62. 16988077 10.1038/oby.2006.153

[93] Hassnain Waqas SF Noble A Hoang AC. Adipose tissue macrophages develop from bone marrow-independent progenitors in Xenopus laevis and mouse. J Leukoc Biol. 2017;102(3):845–55. 28642277 10.1189/jlb.1A0317-082RR PMC5574031

[94] Amano SU Cohen JL Vangala P. Local proliferation of macrophages contributes to obesity-associated adipose tissue inflammation. Cell Metab. 2014;19(1):162–71. 24374218 10.1016/j.cmet.2013.11.017 PMC3931314

[95] Lumeng CN Bodzin JL Saltiel AR. Obesity induces a phenotypic switch in adipose tissue macrophage polarization. J Clin Invest. 2007;117(1):175–84. 17200717 10.1172/JCI29881 PMC1716210

[96] Wakamatsu T Yamamoto S Ito T. Indoxyl sulfate promotes macrophage IL-1beta production by activating aryl hydrocarbon receptor/NF-kappa/MAPK cascades, but the NLRP3 inflammasome was not activated. Toxins (Basel). 2018;10(3):124. 29543732 10.3390/toxins10030124 PMC5869412

[97] Nakano T Katsuki S Chen M. Uremic toxin indoxyl sulfate promotes proinflammatory macrophage activation via the interplay of OATP2B1 and Dll4-Notch signaling. Circulation. 2019;139(1):78–96. 30586693 10.1161/CIRCULATIONAHA.118.034588 PMC6311723

[98] Kim HY Yoo TH Cho JY. Indoxyl sulfate-induced TNF-alpha is regulated by crosstalk between the aryl hydrocarbon receptor, NF-kappaB, and SOCS2 in human macrophages. FASEB J. 2019;33(10):10844–58. 31284759 10.1096/fj.201900730R

[99] Kratz M Coats BR Hisert KB. Metabolic dysfunction drives a mechanistically distinct proinflammatory phenotype in adipose tissue macrophages. Cell Metab. 2014;20(4):614–25. 25242226 10.1016/j.cmet.2014.08.010 PMC4192131

[100] Song SH Oh TR Choi HS. High serum adiponectin as a biomarker of renal dysfunction: results from the KNOW-CKD study. Sci Rep. 2020;10(1):5598. 32221363 10.1038/s41598-020-62465-2 PMC7101406

[101] Stenvinkel P Marchlewska A Pecoits-Filho R. Adiponectin in renal disease: relationship to phenotype and genetic variation in the gene encoding adiponectin. Kidney Int. 2004;65(1):274–81. 14675060 10.1111/j.1523-1755.2004.00370.x

[102] Kim HY Bae EH Ma SK. Association of serum adiponectin level with albuminuria in chronic kidney disease patients. Clin Exp Nephrol. 2016;20(3):443–9. 26445954 10.1007/s10157-015-1173-4

[103] Huang JW Yen CJ Chiang HW. Adiponectin in peritoneal dialysis patients: a comparison with hemodialysis patients and subjects with normal renal function. Am J Kidney Dis. 2004;43(6):1047–55. 15168385 10.1053/j.ajkd.2004.02.017

[104] Martinez Cantarin MP Keith SW Waldman SA. Adiponectin receptor and adiponectin signaling in human tissue among patients with end-stage renal disease. Nephrol Dial Transplant. 2014;29(12):2268–77. 25049200 10.1093/ndt/gfu249 PMC4240178

[105] Alix PM Guebre-Egziabher F Soulage CO. Leptin as an uremic toxin: deleterious role of leptin in chronic kidney disease. Biochimie. 2014;105:12–21. 25010649 10.1016/j.biochi.2014.06.024

[106] Shankar A Syamala S Xiao J. Relationship between plasma leptin level and chronic kidney disease. Int J Nephrol. 2012;2012:269532. 22666590 10.1155/2012/269532 PMC3361181

[107] Sharma K Considine RV. The Ob protein (leptin) and the kidney. Kidney Int. 1998;53(6):1483–7. 9607179 10.1046/j.1523-1755.1998.00929.x

[108] Sharma K Considine RV Michael B. Plasma leptin is partly cleared by the kidney and is elevated in hemodialysis patients. Kidney Int. 1997;51(6):1980–5. 9186891 10.1038/ki.1997.269

[109] Pecoits-Filho R Nordfors L Heimburger O. Soluble leptin receptors and serum leptin in end-stage renal disease: relationship with inflammation and body composition. Eur J Clin Invest. 2002;32(11):811–7. 12423321 10.1046/j.1365-2362.2002.01063.x

[110] Loffreda S Yang SQ Lin HZ. Leptin regulates proinflammatory immune responses. FASEB J. 1998;12(1):57–65. 9438411

[111] La Cava A Alviggi C Matarese G. Unraveling the multiple roles of leptin in inflammation and autoimmunity. J Mol Med (Berl). 2004;82(1):4–11. 14556053 10.1007/s00109-003-0492-1

[112] Cohen G Raupachova J Ilic D. Effect of leptin on polymorphonuclear leucocyte functions in healthy subjects and haemodialysis patients. Nephrol Dial Transplant. 2011;26(7):2271–81. 21216885 10.1093/ndt/gfq731 PMC3164446

[113] Barazzoni R Gortan Cappellari G Zanetti M. Ghrelin and muscle metabolism in chronic uremia. J Ren Nutr. 2012;22(1):171–5. 22200437 10.1053/j.jrn.2011.10.017

[114] Yoshimoto A Mori K Sugawara A. Plasma ghrelin and desacyl ghrelin concentrations in renal failure. J Am Soc Nephrol. 2002;13(11):2748–52. 12397045 10.1097/01.asn.0000032420.12455.74

[115] Buscher AK Buscher R Hauffa BP. Alterations in appetite-regulating hormones influence protein-energy wasting in pediatric patients with chronic kidney disease. Pediatr Nephrol. 2010;25(11):2295–301. 20607302 10.1007/s00467-010-1588-9

[116] Mak RH Cheung W Cone RD. Mechanisms of disease: cytokine and adipokine signaling in uremic cachexia. Nat Clin Pract Nephrol. 2006;2(9):527–34. 16941045 10.1038/ncpneph0273

[117] Guebre-Egziabher F Bernhard J Geelen G. Leptin, adiponectin, and ghrelin dysregulation in chronic kidney disease. J Ren Nutr. 2005;15(1):116–20. 15648019 10.1053/j.jrn.2004.09.015

[118] Yamauchi T Nio Y Maki T. Targeted disruption of AdipoR1 and AdipoR2 causes abrogation of adiponectin binding and metabolic actions. Nat Med. 2007;13(3):332–9. 17268472 10.1038/nm1557

[119] Meacham CE Jeffery EC Burgess RJ. Adiponectin receptors sustain haematopoietic stem cells throughout adulthood by protecting them from inflammation. Nat Cell Biol. 2022;24(5):697–707. 35513711 10.1038/s41556-022-00909-9 PMC9107511

[120] Yamauchi T Kamon J Minokoshi Y. Adiponectin stimulates glucose utilization and fatty-acid oxidation by activating AMP-activated protein kinase. Nat Med. 2002;8(11):1288–95. 12368907 10.1038/nm788

[121] Shimotomai T Kakei M Narita T. Enhanced urinary adiponectin excretion in IgA-nephropathy patients with proteinuria. Ren Fail. 2005;27(3):323–8. 15957550

[122] Friedman JM Halaas JL. Leptin and the regulation of body weight in mammals. Nature. 1998;395(6704):763–70. 9796811 10.1038/27376

[123] Morton GJ. Hypothalamic leptin regulation of energy homeostasis and glucose metabolism. J Physiol. 2007;583(Pt 2):437–43. 17584844 10.1113/jphysiol.2007.135590 PMC2277030

[124] Mak RH Cheung W. Energy homeostasis and cachexia in chronic kidney disease. Pediatr Nephrol. 2006;21(12):1807–14. 16897005 10.1007/s00467-006-0194-3

[125] Mak RH Cheung W Cone RD. Leptin and inflammation-associated cachexia in chronic kidney disease. Kidney Int. 2006;69(5):794–7. 16518340 10.1038/sj.ki.5000182

[126] Cumin F Baum HP Levens N. Leptin is cleared from the circulation primarily by the kidney. Int J Obes Relat Metab Disord. 1996;20(12):1120–6. 8968858

[127] Merabet E Dagogo-Jack S Coyne DW. Increased plasma leptin concentration in end-stage renal disease. J Clin Endocrinol Metab. 1997;82(3):847–50. 9062494 10.1210/jcem.82.3.3817

[128] Cheung W Yu PX Little BM. Role of leptin and melanocortin signaling in uremia-associated cachexia. J Clin Invest. 2005;115(6):1659–65. 15931394 10.1172/JCI22521 PMC1136984

[129] Cheung WW Rosengren S Boyle DL. Modulation of melanocortin signaling ameliorates uremic cachexia. Kidney Int. 2008;74(2):180–6. 18432186 10.1038/ki.2008.150

[130] Markison S Foster AC Chen C. The regulation of feeding and metabolic rate and the prevention of murine cancer cachexia with a small-molecule melanocortin-4 receptor antagonist. Endocrinology. 2005;146(6):2766–73. 15774557 10.1210/en.2005-0142

[131] Arabi T Shafqat A Sabbah BN. Obesity-related kidney disease: Beyond hypertension and insulin-resistance. Front Endocrinol (Lausanne). 2022;13:1095211. 36726470 10.3389/fendo.2022.1095211 PMC9884830

[132] Guebre-Egziabher F Alix PM Koppe L. Ectopic lipid accumulation: a potential cause for metabolic disturbances and a contributor to the alteration of kidney function. Biochimie. 2013;95(11):1971–9. 23896376 10.1016/j.biochi.2013.07.017

[133] Hammoud SH AlZaim I Al-Dhaheri Y. Perirenal adipose tissue inflammation: novel insights linking metabolic dysfunction to renal diseases. Front Endocrinol (Lausanne). 2021;12:707126. 34408726 10.3389/fendo.2021.707126 PMC8366229

[134] Martinez-Garcia C Izquierdo-Lahuerta A Vivas Y. Renal lipotoxicity-associated inflammation and insulin resistance affects actin cytoskeleton organization in podocytes. PLoS One. 2015;10(11):e0142291. 26545114 10.1371/journal.pone.0142291 PMC4636358

[135] Jao TM Nangaku M Wu CH. ATF6alpha downregulation of PPARalpha promotes lipotoxicity-induced tubulointerstitial fibrosis. Kidney Int. 2019;95(3):577–89. 30639234 10.1016/j.kint.2018.09.023

[136] Petreski T Piko N Ekart R. Review on inflammation markers in chronic kidney disease. Biomedicines. 2021;9(2):182. 33670423 10.3390/biomedicines9020182 PMC7917900

[137] Wolf G Hamann A Han DC. Leptin stimulates proliferation and TGF-beta expression in renal glomerular endothelial cells: potential role in glomerulosclerosis [seecomments]. Kidney Int. 1999;56(3):860–72. 10469355 10.1046/j.1523-1755.1999.00626.x

[138] Tarzi RM Cook HT Jackson I. Leptin-deficient mice are protected from accelerated nephrotoxic nephritis. Am J Pathol. 2004;164(2):385–90. 14742244 10.1016/S0002-9440(10)63128-8 PMC1602275

[139] Briffa JF Grinfeld E Mathai ML. Acute leptin exposure reduces megalin expression and upregulates TGFbeta1 in cultured renal proximal tubule cells. Mol Cell Endocrinol. 2015;401:25–34. 25478926 10.1016/j.mce.2014.11.024

[140] Sharma K Ramachandrarao S Qiu G. Adiponectin regulates albuminuria and podocyte function in mice. J Clin Invest. 2008;118(5):1645–56. 18431508 10.1172/JCI32691 PMC2323186

[141] Ohashi K Iwatani H Kihara S. Exacerbation of albuminuria and renal fibrosis in subtotal renal ablation model of adiponectin-knockout mice. Arterioscler Thromb Vasc Biol. 2007;27(9):1910–7. 17626903 10.1161/ATVBAHA.107.147645

